# The Truth about the Milne Treatment

**Published:** 1914-04-04

**Authors:** 


### ISOLATION HOSPITALS AND SCARLET FEVER.

### The Truth about the Milne Treatment.

Some time ago the treatment of scarlet fever by means of 10 per cent. carbolic acid solutions in oil applied to the fauces, together with inunction of eucalyptus oil, as advocated by Dr. Milne, was described in these columns. Since then many trials of this system have been made by independent experts, most of whom have pronounced it disappointing. The originator of the treatment claimed three advantages for it: lessened incidence of complications, lessened duration of the disease, and reduction of the number of "return" cases. Criticisms of the treatment have been chiefly based upon series of cases in which no lessening of complications has been shown. But even if the Milne treatment fails in this respect, it may still be useful in bringing about lessened infectivity and so assisting in the control of epidemics. In the *Edinburgh Medical Journal* Dr. W. Robertson, Medical Officer of Health for Leith, discusses the statistics and experience of scarlet fever during the past three years at the Leith Isolation Hospital. Dr. Milne's treatment was carried out, and great care was also taken to allow all the patients abundance of fresh air and sunshine. With the exception of those suffering from discharges or ulcerated surfaces, the patients are allowed to return home after four to five weeks in hospital even if desquamation is still proceeding—a period dangerously short according to older ideas. Indeed, the average stay of these uncomplicated cases in 1912 is given as 29.8 days. Yet return cases are very infrequent. Complications, however, were not reduced in frequency.

Dr. Robertson points out that the special advantage of the Milne treatment is when home treatment is adopted; he appears to have abandoned it for hospital cases, on the ground that it is unnecessary. He quotes some very interesting figures from his own registers, showing the lessening of infectivity brought about by the Milne method when patients are having domiciliary treatment. These statistics do not cover a sufficiently large number of cases to be accepted as they stand in proof of this; but they do point towards the conclusion that Dr. Robertson draws. The corollary of the success with which the spread of infection can be thus prevented when a case of scarlet fever is being treated at home has been so marked that he pleads for a reconsideration of the routine custom of removing every case of scarlet fever at once to an isolation hospital. He believes that by the Milne treatment the danger of domiciliary treatment to the community can be so far overcome as to render it legitimate.

The expense of isolation hospitals for scarlet fever is, of course, very great; and if it should be found that no risk to the public health is involved by home treatment, very important savings in the rates could be effected. In Leith Dr. Robertson estimates that in one way and another he has saved the ratepayers a penny in the pound per annum over scarlet fever alone; and what is true of Leith could doubtless be applied to other towns and boroughs. The only people likely to be discomposed by this prospect will be the medical officers of the fever hospitals. As they are the experts whose reports have been, on the whole, unfavourable to the Milne treatment, it may be suggested that they have contemplated too fixedly the results in connection with the incidence of complications, and have not paid sufficient attention to infectivity, an aspect which naturally comes more under the gaze of the medical officer of health. It will, at any rate, be of interest to that section of the institutional world which lives in isolation hospitals to watch the result of Dr. Robertson's appeal and the further reports which it is sure to stimulate.

**Mr. George Arthur Harrop, of Hale, Cheshire, calico printer, has left, after certain bequests, the residue of his property upon trust for his sister for life, with remainder as to £500 to the Manchester Eye Infirmary, and the ultimate residue as his said sister may appoint.**

